# Au Revoir

**Published:** 1890-07

**Authors:** W. X. Sudduth


ADVISORY COMMITTEE.

### AU REVOIR.

SOME three years ago, while abroad, we conceived the idea of establishing an international dental journal. The need for a high-class independent journal to represent dentists and dentistry was only too apparent. The matter was broached to quite a number of foreign dentists, and met with such warm expressions of interest that we returned home earlier than we had anticipated in order to present the matter to the dentists in attendance upon the International Medical Congress, to be held in Washington in August, 1887. It was thought best not to make it public at that time, but to use the opportunity to canvass the feasibility of such a movement among those in attendance. It is needless to say that the movement met with favor and took tangible form at that time. During the winter a corps of associate editors, domestic and foreign, was secured, and the matter was brought to the attention of several well-known publishing firms. It was found, however, that it would be impossible to secure that degree of independence of expression necessary to a journal of the character intended if it were owned outside of the profession, and the next step was to form a journal company within its ranks. This movement was set on foot in New York, and met with such universal approval that within two weeks time over ten thousand dollars had been subscribed to put the project on its feet. It was not until then that we fully realized how ripe the times were, and how badly such an organ was needed. Steps were at once taken to organize a publishing company, and the *International Dental Publication Company* was the result. It may not be uninteresting to know that it is chartered under the laws of the State of New Jersey with a capital stock of five thousand dollars,—that amount being considered sufficient to establish the JOURNAL. This stock is owned by over one hundred dentists in active practice, and as much more could be placed if it were considered desirable to do so. The JOURNAL has been a paying investment from the beginning, and is now fully established as the recognized organ of the profession. The task has been no easy one, and had we known just how difficult it would have been, it is doubtful whether we should have gone into it. It was the first effort ever made to organize the dental profession into an association with an organ for its defence. The need of such a movement had long been felt, but the evident lack of *esprit de corps* in the profession had deterred others from making the attempt. This difficulty has, however, been overcome, and dentistry stands to-day fully organized with an organ which has demonstrated its fitness to occupy the position its promoters sought in the first instance to establish for it. It is *international* in character, *independent* in speech, *broad* in spirit, and *high* in tone.

As has been intimated in the announcement by the *Advisory Committee*, with this issue our active duties as Editor and Business Manager close. This step is made necessary by our appointment to a position which seems to give promise of a field of labor more in keeping with our tastes. The active work on the JOURNAL has so absorbed our time for the past two and one-half years that we have been unable to pursue our favorite lines of research in biology. It is with regret that we sever an association which has been so pleasant in the main, but in doing so, we do not go out of the company, our interests will still remain in it, and we shall always be found working for its advancement. The business of the Company will be placed in reliable hands, that will give its financial management their entire attention. This has been considered absolutely essential, as the rapidly-growing business is so absorbing in its demands that no professional man, actively engaged in practice or scientific work, can find time to attend to it. There will be no change in the policy of the JOURNAL. Dr. Truman has, as chairman of the Advisory Committee of the Board of Directors, been so intimately associated with the management of the JOURNAL that the work will go on without a jar, and the INTERNATIONAL will continue to stand in the future, as it has in the past, the fearless voice of the profession. Dr. Truman is too well known to need any word of introduction at our hands. His long experience as teacher and active worker in the profession pre-eminently fits him for the position, and the Company is to be congratulated that he has consented to assume the editorial chair even temporarily, and it is to be hoped that he may find it to his interest to continue in the position.

W. X. SUDDUTH.

